# The Impact of a Mindfulness App on Postnatal Distress

**DOI:** 10.1007/s12671-022-01992-7

**Published:** 2022-09-28

**Authors:** Katie A. Bear, Carol C. Barber, Oleg N. Medvedev

**Affiliations:** School of Psychology, University of Waikato, Hillcrest, Private Bag 3105, 3240 Hamilton, New Zealand

**Keywords:** Mindfulness-based intervention, Mobile phone app, Postnatal mental health, Depression, Stress

## Abstract

**Objectives:**

The present study investigated the effectiveness of an 8-week mindfulness mobile phone app on women’s depression, anxiety, stress and mindful attention/awareness in the postnatal period.

**Methods:**

The study enrolled 99 mothers of a child under 1 year old, and randomly assigned them to intervention (*n* = 49, mean age = 31.11, *SD* = 4.30, years) and control (*n* = 50, mean age = 31.35, *SD* = 5.29, years) groups. Multiple regression examined intervention effects on depression, anxiety, stress and mindful attention/awareness measured post-intervention and at 4-week follow-up, controlling for the baseline and post-intervention measurement of the specific outcome, respectively.

**Results:**

The intervention group showed significant decreases in depression, anxiety and stress levels and an increase of mindful attention/awareness post-intervention compared to the control group, with medium to large effect sizes after controlling for effects of corresponding variables at baseline. The intervention group showed further decrease in depression and stress levels and an increase in mindful attention/awareness at 4 weeks post-intervention compared to the control group, with small to medium effect sizes, after controlling for effects of corresponding variables at post-intervention.

**Conclusions:**

The outcomes of the study suggest that delivery of mindfulness via smartphones could be a viable and affordable resource for reducing postnatal depression, anxiety and stress.

Having a child is a significant life shift experienced by new parents. This involves changes in roles, responsibilities and expectations within families and their intimate partner relationships, while at the same time developing a bond with their infant. While there might be expectations that parenthood will be a time of joy and fulfilment, there are substantial stressors that accompany this period that place mothers at risk for emotional and cognitive distress (Yelland et al., 2010).

There are common stressors that accompany the postnatal period, which refers to the first year after birth. The most common stressors are recovering from birth, challenges with breastfeeding and bottle-feeding, lack of sleep, changes in hormones, coping with an unsettled baby, and disturbances in parent and infant bond (Milgrom et al., 2007). These stressors manifest in addition to ongoing household duties, and, for many mothers, resuming work outside the home (Biaggi et al., 2016). Due to these stressors, it is no surprise that there is a substantial body of research evidence demonstrating that a subset of mothers experience a range of mental health problems in the first year after a child’s birth, including anxiety, depression and stress (Camisasca et al., 2021; O’Hara & Swain, 2009; Yelland et al., 2010). To decrease the likelihood of these stressors leading to adverse mental health outcomes, there is a real need for accessible and effective treatments.

Postnatal depression (PND) is a common and serious postpartum mental disorder, affecting an estimated 10–20% of birthing parents during the postpartum period (Corbally & Wilkinson, 2021; Woody et al., 2017), with a disproportionately higher prevalence for those with middle-low income compared to high income (Woody et al., 2017). Beck et al. (2011) found that up to 42% of women experienced elevated postpartum depressive symptoms which did not meet a clinical threshold. However, it has been suggested that less than 50% of women who experience postnatal depressive symptoms will seek any formal assistance (Signal et al., 2017). Women who develop PND have an increased risk of experiencing further episodes of depression, and up to a 41% rate of experiencing PND again with a subsequent child (Cooper & Murray, 1998; Tharwat et al., 2018).

The impact of postnatal depression extends beyond the parent, with associated negative repercussions for the child (Heron et al., 2004). PND can lead to a disruption to healthy mother-infant bonding (Kerstis et al., 2015). Mothers with PND also report significantly higher rates of problematic patterns or perceptions of their infants’ behaviour in relation to feeding, crying and sleeping (Trevarthen & Aitken, 2001), and infants of mothers with PND have a higher risk of delayed motor, neurological, cognitive, language and emotional development, as well as displaying higher rates of distress and avoidance behaviour (O’Connor et al., 2002; Weissman et al., 2006). These adverse consequences can continue well past infancy and into adulthood (Weissman et al., 2006).

Postnatal anxiety is common and frequently co-occurs with postnatal depression, yet it is often neglected in studies of pregnancy and the postnatal period (Heron et al., 2004). Problematic levels of anxiety in the postnatal period have an estimated prevalence of 5–33% (Leach et al., 2017). Those experiencing postnatal anxiety can show impaired decision-making and reduced social functioning (Highet et al., 2014). In some instances, mothers report having feelings of panic, sometimes accompanied with severe and recurrent intrusive thoughts that often focus on their baby (Dennis et al., 2017). Evidence for adverse outcomes for the infants of anxious mothers is growing. Postnatal maternal anxiety has been linked with emotional and behavioural maladjustment of the infant (Bauer et al., 2015; Bekkhus et al., 2011; O’Connor et al., 2002; Rees et al., 2019).

Stress is a distinct adverse emotional state involving heightened arousal and impaired function (Lovibond & Lovibond, 1995). Significantly elevated stress levels in the postpartum period have been shown to affect approximately 40% of women in developed nations (Anniverno et al., 2013; Beck et al., 2011; Miller et al., 2006). Postnatal depression, anxiety and stress are all unique and separate experiences of distress that can interact and influence each other. Meta-analytic reviews have consistently identified stress within the postnatal period to be a significant risk factor that can contribute to the development of and precipitate PND and anxiety (Bernazzani et al., 1997; O’Hara & Swain, 2009). Research examining the course of postnatal depression suggests that anxiety may develop into depression as a result of the inability to manage stress (Heron et al., 2004). Another study found the symptoms of depression to be linked to the degree of stress (Terry et al., 1996).

Mindfulness-based interventions (MBIs) are structured psychological interventions that incorporate mindfulness practice. MBIs are designed with the intent to teach the individual to cultivate mindfulness and incorporate its practice into everyday life (Kabat-Zinn, 2003). They have been found beneficial for many conditions and issues, such as decreasing stress (Economides et al., 2018), anxiety (Quinones & Griffiths, 2019), depression (Flett et al., 2018), aggression (Shastri et al., 2017), compulsive internet use (Quinones & Griffiths, 2019) and job strain (Bostock et al., 2019) and increasing well-being (Howells et al., 2016), resilience (Flett et al., 2018), self-compassion (Dunn et al., 2012) and perceptions of workplace social support (Bostock et al., 2019). Dispositional mindfulness has been found to be inversely predictive of perinatal distress, with lower levels of mindfulness associated with elevated levels of depression, stress and anxiety (Kalmbach et al., 2020; Khan & Laurent, 2019).

There is growing evidence that MBIs have the potential to reduce depression, anxiety and stress for mothers in the perinatal period. A meta-analysis and systemic review of the effectiveness of MBIs in the perinatal period revealed significant reduction of depression, anxiety and stress concurrent with increases in mindfulness skills (Taylor et al., 2016). Of the studies assessed in this review, the majority (94%) were group-administered, in-person interventions. A recent meta-analysis of the effectiveness of MBIs delivered in the prenatal period also found them to be effective in reducing depression, anxiety and stress in pregnant participants (Corbally & Wilkinson, 2021). Moreover, MBIs have been shown to further improve well-being outcomes when combined with pharmacological treatment (Shulman et al., 2018). Notably, research has shown that that 8-week MBIs produced significant reductions in postnatal participants’ overall distress, depression and stress at follow-ups ranging from 8 weeks to 18 months (Ahmadpanah et al., 2018; Dimidjian et al., 2016; Felder et al., 2018; Pan et al., 2019). Even further decreases of PND have been seen at 3-month follow-up (Shulman et al., 2018).

Mindfulness refers to the ability to maintain attention to and awareness of present-moment experiences, emotions, thought and physical sensations as they unfold while maintaining a non-judgmental attitude (Kabat-Zinn, 2003). In the clinical context, there are various operational definitions, but the general consensus is that mindfulness levels can be described as the non-judgmental observation of the continuing surge of internal and external stimuli as they occur (Feng et al., 2018). Mindfulness levels can be measured as state and dispositional factors. State mindfulness is the quality of mindful presence at a given moment, or within a narrow window of time (e.g. the past 5 min), whereas dispositional or trait mindfulness refers to the general, cross-situational frequency of mindful states over time (Brown & Ryan, 2003).

In perinatal research on the effects of mindfulness, levels of stress, depression and anxiety are often studied separately, but rarely together (Felder et al., 2016; Fontein-Kuipers et al., 2014; Khan & Laurent, 2019; Townshend & Caltabiano, 2019; Townshend et al., 2018). It has been argued that research within the perinatal period frequently labels coexisting symptoms of postpartum depression and anxiety as PND without differentiating between these experiences (Miller et al., 2006). Because these factors are separate and affect the individual uniquely, research measuring all three symptoms is important to better understand how these separate experiences distinctively respond to interventions. Within the literature reviewed, one study was located that investigated the effect of an 8-week mindful parenting intervention on levels of stress, depression and anxiety, using a pre-post design method. They assessed symptoms of depression, anxiety and stress via the DASS21 at pre-test, post-test and 8-week follow-up (Potharst et al., 2022). They found significant reductions in all outcome variables at both measurement points apart from anxiety at the 8-week follow-up.

Evidence has showed that in-person, group-based MBIs can have a significant impact on perinatal well-being. However, Taylor et al. (2016) noted that retention rates in these interventions average about 75%, highlighting barriers to accessing this type of programmes. There are accessibility barriers for mothers that may limit the ability to physically attend face-to-face treatment. Interviews have revealed structural barriers to services to be greater obstacles than knowledge and attitudinal barriers, with the greatest structural barriers reported being lack of time, finances, transport and childcare (Goodman, 2009; O’Mahen & Flynn, 2008).

In response to overcoming these structural barriers, MBIs delivered on an application (app) via a smartphone are growing in popularity (Marshall et al., 2020). MBIs delivered via app (aMBI) have been shown to significantly reduce anxiety and depressive symptoms (Gál et al., 2021), irritability, affect and stress resulting from personal vulnerability, external pressure (Economides et al., 2018) and compulsive internet use (Quinones & Griffiths, 2019). In addition, they have been found to increase job-related well-being (Bostock et al., 2019), resilience (Flett et al., 2018) and positive affect (Howells et al., 2016). There has been little research examining the effectiveness of aMBIs in the postnatal period, highlighting a gap in literature. A recent scoping review of mobile-based psychological interventions for perinatal depression and anxiety only identified one study of the effectiveness of aMBI, from the 22 studies reviewed (Hussain-Shamsy et al., 2020).

The current study sought to investigate the effectiveness of an 8-week aMBI aiming to reduce experiences of depression, anxiety and stress in mothers during the postnatal period. It was hypothesised that depression, anxiety and stress levels in mothers who participate in the 8-week app-based mindfulness intervention would be significantly reduced compared to the active control condition, with further improvements seen at 4-week follow-up. It was also hypothesised that levels of mindful attention/awareness in those who participated in the 8-week mindfulness intervention would be significantly increased compared to the control condition, with further improvements seen at 4-week follow-up.

## Method

### Participants

Recruitment of participants was conducted through social media posts on pages that targeted mothers, primarily focusing on New Zealand mothers, but with some dissemination in Australian groups. Recruitment took place between October 14, 2019, and March 25, 2020. Inclusion criteria consisted of (a) being the mother of a child aged 0–12 months and (b) access to a smartphone that can download applications. New Zealand went into a strict lockdown on March 25, 2020, in response to the COVID-19 pandemic. Due to this major disruption and its unknown effects on social and psychological functioning, recruitment was discontinued at that point.

A total of 99 participants enrolled in the study and were randomly assigned to treatment and control groups. There were 49 participants assigned to the experimental group, with 50 participants assigned to the control group. Demographic characteristics of the mothers for each condition are shown in Table 1. Preliminary analysis confirmed that there were no statistically significant differences between the two groups at baseline for age, mindful attention/awareness and distress outcome variables.

**Table 1:** Participant demographics

Characteristic	Overall sample		Smiling Mind	Control
	*n* = 99	*n* = 49		*n* = 50
Age mean (*SD*)	31.16 (4.83)	31.11 (4.30)		31.35 (5.29)
Age range	21–45	22–39		21–45
Child age (months)	4.03 (3.24)	3.50 (2.5)		4.49 (7.10)
Child age range (months)	0–12	0–10		0–12
Ethnicity				
New Zealand Māori	7 (7%)	6 (12%)		1 (2%)
Pākehā/European	78 (79%)	35 (71%)		43 (86%)
Asian	2 (2%)	2 (4%)		0 (0%)
Other	12 (12%)	6 (12%)		6 (12%)
Relationship status				
Married/de facto living together	89 (90%)	43 (88%)		46 (92%)
Married/de facto not living together	2 (2%)	2 (4%)		0 (0%)
Separated	2 (2%)	1 (2%)		1 (2%)
Single	6 (6%)	3 (6%)		3 (6%)
Currently using a mobile app for perinatal support				
Yes	30 (30%)	16 (33%)		14 (28%)
No	69 (70%)	33 (67%)		36 (72%)
Country of residence				
New Zealand	93 (94%)	46 (94%)		47 (94%)
Australia	6 (6%)	3 (6%)		3 (6%)
Practiced mindfulness				
More than a year ago	22 (22%)	10 (20%)		12 (24%)
6–12 months ago	12 (12%)	5 (10%)		7 (14%)
3–6 months ago	14 (14)	11 (22%)		3 (6%)
Never practised	48 (48%)	21 (43%)		20 (40%)
Currently practising	3 (3%)	2 (4%)		1 (2%)

### Procedures

**Fig. 1:**
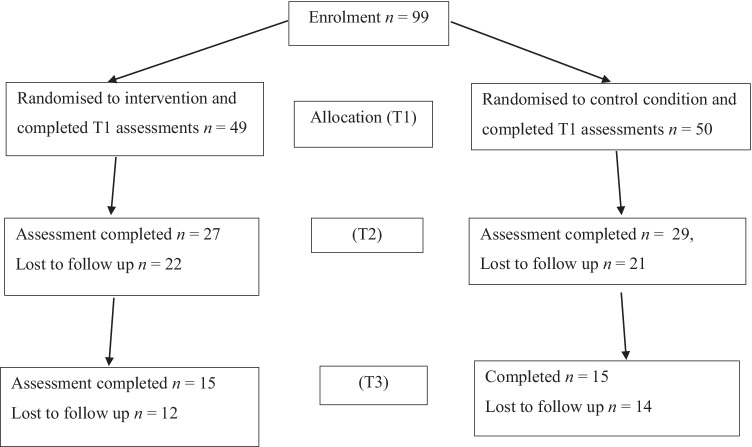
Flow diagram of participant involvement

The study used a pre- to post-RCT design that tested the effect of a mindfulness app (Smiling Mind) on distress levels in the postnatal period, relative to a control app (Baby + Tracker). This study required women to use an app for 8 weeks, and respond to a battery of assessment tools to measure baseline (T1), post-intervention (T2) and 4-week follow-up scores (T3). See Fig. 1 for flow diagram. The trial was not preregistered.

There were a total of four formal withdrawals from the study citing inadequate time to use the app (2), the app being incompatible with their phone (1) and no reason given (1). A further 38 dropped out of the study by not completing the post-intervention (T2) survey. A total of 56 participants completed the T2 survey, with 27 participants in the experimental group and 29 participants in the control group. A further 27 did not complete the 4-week follow-up (T3) survey, resulting in a total of 30 participants completing both the intervention and follow-up survey with 15 participants in the experimental group and 15 participants in the control group.

Using the online survey platform Qualtrics, participants read the information sheet and gave informed consent by confirming they had read the information provided and were ready to begin the study. The participants then completed the questionnaires and were randomly assigned to either the experimental or control condition. Participants were instructed to download the applicable app and use it for 8 weeks. Participants in the experimental condition were encouraged to complete one session per day (session duration in minutes: *M* = 9.20), with the request to complete a minimum of three sessions per week. The control condition was encouraged to use the smartphone application that offers a combination of postnatal support and information unrelated to mindfulness for about 10 min per day, with the request to use it a minimum of three sessions per week. Participants were sent out a weekly SMS reminding them to use the app. On completion of the 8 weeks and at 4-week follow-up, participants were sent a link to complete measures of distress and mindful attention/awareness. If surveys were not completed, then up to two reminder SMS were sent. Participants were debriefed on the study after the final scale was completed.

#### Intervention

##### Smiling Mind Application

Smiling Mind (Smiling Mind, 2021) is a smartphone application that offers hundreds of hours of guided and unguided mindfulness meditation practices across several different mindfulness programmes. Smiling Mind is available free of charge on iOS and Android platforms and through the Smiling Mind website. Participants randomised to the Smiling Mind condition were instructed to download the app and use the ‘Mindfulness Foundations’ programme. The ‘Mindfulness Foundations’ programme features 10 modules over 41 sessions. The meditation sessions vary in duration from 1 to 43 min (*M* = 9.20). The ten modules cover the topics of the following: the Breath, Sound and Taste, Thoughts, Emotions, Everyday Mindfulness, Curiosity and Beginner’s Mind, Stress, Sleep and Gratitude, Relationships and Mindful Listening. The ‘Mindfulness Foundations’ programme is an introduction to mindfulness and not aimed specially for the postnatal period.

#### Control Condition

##### Baby + Tracker Application

Baby + Tracker (Philips Electronics UK, 2021) is a smartphone application that offers a combination of postnatal support and information, tracking infant progress, milestones and daily routine, and capturing memorable moments. Baby + Tracker is available free of charge on iOS and Android platforms. The support and information component includes information such as week by week development information, parenting and breastfeeding guides. The tracking of infant progress and milestones and daily routine components includes a baby growth and sleep tracker, milestones log and health tracker. The capture memorable moments component gives options to store and retrieve photos and videos of the infant.

### Measures

Participants completed three scales to assess levels of distress and mindful attention/awareness, as well as a demographic questionnaire. Demographic information collected included the age of participant and infant, ethnicity, relationship status, country of residence, history of practising mindfulness and current use of support apps for postpartum support.

#### Depression Anxiety and Stress Scale Short Form (DASS21)

The DASS21 (Lovibond & Lovibond, 1995) is a 21-item self-report measure that assesses symptoms of depression, anxiety and stress. Using a 4-point Likert-type scale ranging from *0* (*did not apply to me at all*) to *3* (*applied to me very much, most of the time*), participants were asked to indicate how much each statement applied to them over the past 7 days. The DASS21 produces subscales of Depression (DASS-D), Anxiety (DASS-A) and Stress (DASS-S) by summing the scores for each of the seven relevant items for each subscale. Scores range from 0 to 21 for each subscale, with higher scores indicating higher symptomatology. The raw score of the DASS21 were used for interpretation. Scores can be categorised into normal, mild, moderate, severe and extremely severe. The DASS21 was developed in Australia and is commonly used in Australasia (Crawford et al., 2011). The depression subscale does not include somatic symptoms and therefore is appropriate for use in postnatal assessment (Barber & Steadman, 2018). The DASS21 has been previously shown to have high internal consistency for all subscales: DASS-D (*α* = 0.94; McDonald’s *ω* = 0.86), DASS-A (*α* = 0.87; McDonald’s *ω* = 0.82) and DASS-S (*α* = 0.91; McDonald’s *ω* = 0.88; Antony et al., 1998; Osman et al., 2012). Internal consistency in the current study was high for all subscales: DASS-D (*α* = 0.95), DASS-A (*α* = 0.93) and DASS-S (*α* = 0.90).

#### Mindful Attention Awareness Scale (MAAS)

The MAAS (Brown & Ryan, 2003) is a 15-item scale that measures core characteristics of mindful attention/awareness. Using a 6-point Likert-type scale ranging from *1* (*Almost always*) to *6* (*Almost never*), participants were asked to indicate how frequently or infrequently they had each experience. The scores range from 0 to 90, with higher scores indicating higher levels of mindful attention/awareness. The MAAS is widely used in studying mindfulness interventions and has been previously shown to have high internal consistency (*α* = 0.92; McDonald’s *ω* = 0.93; González-Blanch et al., 2022). Internal consistency in the current study was high (*α* = 0.94).

### Data Analyses

Statistical analyses were conducted using IBM SPSS v. 26 for Windows. Preliminary analyses examined baseline group differences and found no statistically significant difference between the groups on any measure. The hypotheses and research questions were subsequently examined by conducting separate sets of hierarchical multiple regression while controlling for baseline levels of outcome variables. The presented descriptions of the magnitude of the effects of *R*^2^ follow the general definitions of *R*^2^ of 0.02 as small, 0.15 as medium and 0.26 as large (Cohen, 1988). Statistical significance was also evaluated and significance levels of less than 0.05 are reported as significant.

The target sample size was determined based on previous research by Taylor et al. (2016), who conducted a meta-analysis of studies on mindfulness-based interventions on distress in the perinatal period, yielding average medium effect sizes of Hedge’s *g* = 0.47 for depression, 0.36 for anxiety and 0.51 for stress. Minimum required sample size for regression analyses (*n* = 52) was determined using G*Power based on 85% certainty to detect a medium effect size (*R*^2^ = 0.15) under *p* = 0.05 (Faul et al., 2007). Regression analyses were used in order to maximise statistical power while still allowing to control for initial level on the outcome variables.

Tests of statistical assumptions were independently conducted for each analysis at post-intervention and follow-up and are only reported where violations of these assumptions were observed. For the regressions of intervention on DASS-depression, DASS-anxiety and DASS-stress, an inspection of distributions indicated there was a positive skew on DASS-depression, DASS-anxiety and DASS-stress. Square root transformations resulted in normal distributions; therefore, the transformed data for these variables were used for inferential statistics. The remaining variables were normally distributed and free from univariate outliers.

Inspection of the normal probability plot of standard residuals as well as the scatter plot of standardised residuals against standardised predicted values indicated that assumptions of normality, linearity and homoscedasticity of residuals were met. There was no evidence of multicollinearity, as assessed by tolerance values greater than 0.1. There were no studentised residuals greater than ± 3 standard deviations, leverage values greater than 0.2 and values for Cook’s distance above 1. Prior to calculating *r* of the associations between mindful attention/awareness and distress levels, the assumptions of normality, linearity and homoscedasticity were assessed. Visual inspection of the normal Q-Q plots of each variable confirmed that both were normally distributed.

To check for differential dropout, a comparison was conducted between the participants that completed and dropped out at T2, and those that completed and dropped out at T3 on MAAS, DASS-depression, DASS-anxiety and DASS-stress subscales, age of child and age of participant. Two significant differences were found at T2, suggesting that completers scored higher than dropouts on initial ratings of DASS-depression and DASS-anxiety (DASS-D, *F*(1, 56) = 4.91, *p* = 0.03, completers *M* = 6.07, *SD* = 6.3, drop outs *M* = 3.65, *SD* = 3.84; DASS-S, *F*(1, 56) = 8.21, *p* = 0.005, completers *M* = 9.80, *SD* = 5.35, drop outs *M* = 7.00, *SD* = 3.84).

## Results

Descriptive statistics for the outcome variables by condition at T1, T2 and T3 for all participants who completed the assessments are presented in Table 2. At the T3 assessment, all participants were asked if they were still using the applicable app. Sixty percent (9/15) of the intervention group participants that completed T3 and 13% (2/15) of the control group participants that completed T3 reported they were still using the app. Treatment adherence data was collected at T2 and T3. At T2, 90% (44/49) of the intervention group and 86% (43/50) of the control group participants reported 3 ≥ sessions per week, 6% (3/49) of the intervention group and 4% (2/50) of the control group participants reported 1–2 sessions per week, and 4% (2/49) of the intervention group and 10% (5/50) of the control group participants reported ≤ 1 session per week. At T3, 93% (14/15) of the intervention group and 93% (14/15) of the control group participants reported 3 ≥ sessions per week, 7% (1/15) of the intervention group and 0% (0/15) of the control group participants reported 1–2 sessions per week, and 0% (0/15) of the intervention group and 7% (1/15) of the control group participants reported ≤ 1 session per week.

**Table 2:** Means and standard deviations of outcome measures by condition by time (T1-T2-T3)

	MI (*n* = 49)	CC (*n* = 50)	MI (*n* = 27)	CC (*n* = 29)	MI (*n* = 15)	CC (*n* = 15)
	T1		T2		T3	
DASS-D	6.26 (4.37) ^a^22%	6.35 (3.95) ^a^22%	3.52 (3.84) ^a^15%	5.59 (4.26) ^a^24%	3.58 (2.31) ^a^7%	6.5 (3.28) ^a^20%
DASS-A	4.57 (2.13) ^a^25%	5.17 (2.96) ^a^26%	3.07 (3.49) ^a^19%	5.13 (3.70) ^a^27%	3.07 (2.29) ^a^13%	4.73 (3.60) ^a^27%
DASS-S	8.24 (4.57) ^a^24%	8.38 (5.47) ^a^24%	6.19 (4.41) ^a^15%	8.37 (3.96) ^a^24%	5.53 (2.64) ^a^7%	7.47 (3.67) ^a^27%
MAAS	3.28 (1.23)	3.12 (1.11)	4.14 (1.32)	3.28 (1.07)	4.44(1.02)	3.27 (1.01)

*MI*, mindfulness intervention; *CC*, control condition; *DASS-D*, depression subscale; *DASS-A*, anxiety subscale; *DASS-S*, stress subscale. ^a^Percentage of participants scoring in the mild range or higher

Several linear regression models were tested with intervention (vs control) as a predictor variable and outcomes of T2 depressive (DASS-D-T2), anxiety (DASS-A-T2), stress (DASS-S-T2) and mindful attention/awareness levels (MAAS-T2), at post-intervention. The mindfulness intervention demonstrated a large effect on DASS-D-T2, accounting for 45% of variance, large effect on DASS-A-T2, accounting for 41% of variance, a large effect on DASS-S-T2, accounting for 56% of variance, and a large effect on MAAS-T2, accounting for 63% of variance These findings were all statistically significant (Table 3).

**Table 3:** Summary of the regression analyses examining intervention effects of the mindfulness intervention on depression, anxiety, stress and mindful attention-awareness levels at T2 (*n* = 56)

	*B*	*β*	*R*^2^	*F*	*p*
Outcome: DASS-D-T2					
Mindfulness intervention	4.85	0.63	0.45	35.30	< 0.001
Outcome DASS-A-T2					
Mindfulness intervention	4.38	0.72	0.41	56.38	< 0.001
Outcome: DASS-S-T2					
Mindfulness intervention	6.75	0.75	0.56	68.81	< 0.001
Outcome: MAAS-T2					
Mindfulness intervention	− 33.63	− 0.80	0.63	90.15	< 0.001

Several linear regression models were tested with intervention (vs control) as a predictor variable and outcomes of T2 depressive (DASS-D-T3), anxiety (DASS-A-T3), stress (DASS-S-T3) and mindful attention/awareness levels (MAAS-T2), at the 3-month follow-up. The mindfulness intervention demonstrated a large effect on DASS-D-T3, accounting for 48% of variance, large effect on DASS-A-T3, accounting for 27% of variance, a large effect on DASS-S-T3, accounting for 53% of variance, and a large effect on MAAS-T3, accounting for 62% of variance. These findings were all statistically significant (Table 4).

**Table 4:** Summary of the regression analyses examining intervention effects of the mindfulness intervention on depression, anxiety, stress and mindful attention-awareness levels at T3 (*n* = 30)

	*B*	*β*	*R*^2^	*F*	*p*
Outcome: DASS-D-T3					
Mindfulness intervention	4.27	0.70	0.48	26.02	< 0.001
Outcome DASS-A-T3					
Mindfulness intervention	2.46	0.52	0.27	10.53	< 0.001
Outcome: DASS-S-T3					
Mindfulness intervention	5.87	0.73	0.53	37.69	0.003
Outcome: MAAS-T3					
Mindfulness intervention	− 32.93	− 0.79	0.62	91.34	< 0.001

A percentage of participants scored in the mild and higher above depression, anxiety and stress symptoms at all time points in both conditions. The percentage decreased after the intervention and at 4-week follow-up in the intervention but not in the control group (Table 2).

## Discussion

This study examined the effectiveness of participation in the 8-week app-based mindfulness course, compared to an active control, on distress and mindful attention/awareness levels during the postpartum period. The results demonstrated that the app-based mindfulness intervention significantly decreased depression, anxiety and stress and increased mindful attention/awareness post-intervention and at the 4-week follow-up, all showing large effect sizes. These findings suggest that delivery of mindfulness via smartphones could be beneficial for reducing postnatal depression, anxiety and stress while enhancing mindful attention and awareness. Overall, these results are consistent with the findings of previous studies in which an 8-week face-to-face mindfulness intervention resulted in significantly lower levels of depression, anxiety and stress (Fontein-Kuipers et al., 2014; Potharst et al., 2017, 2022).

Previous research with women with postnatal depression has found an 8-week face-to-face mindfulness intervention paired with pharmacological treatment resulted in significantly lower levels of depression than pharmacological treatment alone (Ahmadpanah et al., 2018). These findings suggest that women prescribed medication for postnatal depression could benefit from concurrently using an app that supports mindfulness such as the Smiling Mind app and merits further investigation.

The positive effect of the mindfulness intervention on stress levels is consistent with research by Potharst et al. (2017) showing 8 weeks of mindfulness classes resulted in significant reductions on levels of postnatal stress. The postnatal period has been found to be characterised by increased levels of stress (Anniverno et al., 2013; Beck et al., 2011; Miller et al., 2006); however, depression and anxiety have received much more attention in the literature on interventions in the postnatal period (Felder et al., 2016; Fontein-Kuipers et al., 2014; Khan & Laurent, 2019; Townshend & Caltabiano, 2019; Townshend et al., 2018). This neglect of stress measurements results in the paucity of information around the effect of interventions on subjective stress. Elevated stress is more prominent in the perinatal period than depression and anxiety, affecting 40% of women in developed nations (Anniverno et al., 2013; Beck et al., 2011; Miller et al., 2006). Mothers in the postnatal period may experience high levels of stress without having high levels of depression or anxiety. This study suggests that the use of a mindfulness app can decrease levels of stress in the postnatal period.

The intervention had a large effect on anxiety, which is consistent with published literature, which found that 8-week MBIs resulted in significant decreases in levels of postpartum anxiety (Fontein-Kuipers et al., 2014; Shulman et al., 2018; Taylor et al., 2016). Anxiety can be very disruptive during the postnatal period, so an accessible, effective intervention that addresses it in addition to helping to manage stress and mood is promising and merits further investigation.

The percentage of participants scoring in the mild and higher above depression, anxiety and stress symptoms decreased after the intervention and at 4-week follow-up in the intervention but not in the control group. The trends in the current study show the strongest effects on stress, followed by depression, with the least impact being on anxiety. These patterns of effectiveness are consistent with the meta-analysis by Taylor et al. (2016), showing the same pattern of effectiveness of MBI on stress, depression and anxiety.

A significant and large increase in mindful attention/awareness levels was observed at both post-intervention and the 4-week follow-up for the treatment group. Meta-analytic reviews found that MBIs that failed to increase levels of mindfulness were ineffective in decreasing depression and stress in the perinatal period (Corbally & Wilkinson, 2021; Taylor et al., 2016). The similarities in the findings of the current study and previous research suggest that MBIs delivered face-to-face or via an app may result in similar mechanisms of change, with increases in mindfulness resulting in positive outcomes.

Recent systematic reviews and meta-analyses showed that mobile-based psychological interventions can be effective to prevent depression, anxiety and stress in the general population (Rigabert et al., 2020; Sander et al., 2016), but the evidence is scarce concerning outcomes in the postnatal period. The positive findings of this study suggest that app-based mindfulness interventions may be as effective as face-to-face MBI, with the additional benefit of reducing the barriers to physically attending interventions.

The delivery method of using the app and the free cost mean this intervention can be done in private, which is accessible to anyone with a smartphone, with the only costs being data to download and use the app. This overcomes the barriers to treatment of affordability, transportation, stigma and childcare issues (Ashford et al., 2018; Goodman, 2009; O’Mahen & Flynn, 2008), and may help overcome the barrier to treatment of lack of time (Ashford et al., 2018; Goodman, 2009; O’Mahen & Flynn, 2008). With regard to sustained improvement after the intervention, we observed a continuation of large treatment effects on all factors.

The improvements on depression, anxiety stress and mindful attention/awareness levels continued after the end of the formal intervention; 60% of those who responded at T3 were continuing to use the aMBI app (versus 13% of controls), so this may be an effect of continuing mindfulness practice, rather than true post-intervention change. In the Shulman et al. (2018) study of an in-person MBI that found even further improvements in levels of mindfulness and depression after the intervention, the participants were encouraged to practise mindfulness at home (Shulman et al., 2018). However, Shulman et al. did not report whether participants were practising at home after the intervention ceased. The app for the current mindfulness intervention offered a variety of different mindfulness sessions, rather than being a specific 8-week mindfulness course. This does highlight an opportunity to investigate what characteristics of mindfulness programmes or sessions (e.g. length of sessions, guided vs. unguided, gamification/reinforcement) are associated with continued practice.

The dropout rate for the present study was 43% at T2. Although this is substantial, this rate is not unusual compared to a similar app-based 8-week MBCT intervention aimed at postpartum women, which recorded a dropout rate of 92% at post-intervention (Sun et al., 2021). In the current study, the apps used were chosen based on high user ratings, and this may have contributed to higher retention. In addition, the weekly reminder SMS text messages may have influenced participation. In terms of differential dropout, there were some notable differences between the participants that dropped out and those that completed the interventions. Participants that completed the interventions had significantly higher self-reported levels of depression and stress at the initial assessment point. This suggests that people with depression may be more motivated to complete MBI in app format than mothers who are feeling less distress.

To the best of our knowledge, there is lack of research investigating the effect of an aMBI on mothers’ depression, anxiety and stress levels in postnatal period. The randomised assignment and use of an active control are central strengths of the study. Meta-analytic reviews on MBI in the perinatal period highlighted a lack in rigorous methods and consistency in intervention and outcome measures. One issue frequently highlighted was lack of randomised controlled trials; even in those with control groups, most were assigned to treatment as usual or waitlist (Corbally & Wilkinson, 2021; Taylor et al., 2016). The postnatal period may show a trend of decreased distress over time even without treatment (Takehara et al., 2020), so a control group is particularly important to minimise the chance of overstating gains. Another strength was measurement of depression, anxiety and stress as separate experiences. The segregation of the components of distress is important to be able to investigate treatment effects on differing negative emotions. This allows recommendations of interventions that are suitable for the particular individual’s type of emotional distress.

### Limitations and Directions of Future Research

One major challenge this study faced was the context of the COVID-19 pandemic that was starting to escalate during the time many of the women in this study were participating in the intervention. This affected the sample size and may have affected levels of stress and anxiety. Another limitation was the dropout rate of the present study, of 43% at T2 and 70% at T3. This poses a threat to the validity of results as participants that completed the intervention may differ from the participants that dropped out, and may have benefited more from the intervention. A larger sample size would allow for more detailed evaluation of patterns of change, and more confidence to detect small-sized effects. It is possible that the minimal impact on anxiety found in this study may be in part related to the overall context of high anxiety, but this cannot be examined with the data collected. It is notable that significant effects were found even in the context of this very stressful and isolating context.

A limitation of using multiple self-report measures, which potentially increases the risk of common methods bias, should also be acknowledged. The DASS21 and MAAS require the participants to report their own perceptions, which can be influenced by bias such as priming effects and social desirability, leading to spurious effects as a result of the measurement instruments rather than to the constructs being measured (Podsakoff et al., 2012).

Another limitation of this study is the assessment of mindfulness using the MAAS scale, which is not capturing all relevant facets of mindfulness. For instance, psychometric research has established mindfulness as a multidimensional construct consisting of distinct facets such as observing, describing, acting with awareness, nonreacting and non-judging (Baer et al., 2006). Brown and Ryan (2003) have suggested the MAAS scale more accurately records acting with awareness rather than true mindfulness. A disadvantage of the unidimensional MAAS is that it only allows investigation of mechanisms involved in acting with awareness, which neglects a comprehensive investigation of the overarching mindfulness.

In the initial assessment, participants that completed the intervention reported higher levels of depression and stress than those that dropped out. This indicated those with higher levels of depression and stress may be more likely to persist with this kind of intervention. It would be worth investigating which aspects of mobile applications are related to participants continuing their use.

The current intervention is both remote and autonomous, and relied on participants to report their estimated usage. It would be beneficial to implement tracking of the meditation sessions to improve accuracy. Gathering more precise information into the usage of sessions could give some further insight regarding dropouts, optimal number of sessions for maximum efficacy and optimal session times for retention.

The current study’s inclusion criteria were exclusively targeted at mothers. This is consistent with much of the previous research on mindfulness in the perinatal period, but it would be beneficial to extend research to include the experience of new fathers, who are coping with increased levels of transition and stress at this time, and are often neglected in research on the perinatal mental health (Wong et al., 2016).

App-based mindfulness interventions are a promising tool to support new parents in the complex, stressful, but also rich and rewarding process of transition to parenthood. This study has demonstrated that a freely available mindfulness app can have a substantial impact on levels of depression, anxiety and stress for mothers of infants, and thus constitutes a viable option for promoting well-being in early parenthood.

## Data Availability

The data that support the findings of this study are openly available in Open Science Framework at DOI 10.17605/OSF.IO/QPUZW.
